# Classification of Dried Strawberry by the Analysis of the Acoustic Sound with Artificial Neural Networks

**DOI:** 10.3390/s20020499

**Published:** 2020-01-16

**Authors:** Krzysztof Przybył, Adamina Duda, Krzysztof Koszela, Jerzy Stangierski, Mariusz Polarczyk, Łukasz Gierz

**Affiliations:** 1 Institute of Food Technology of Plant Origin, Faculty of Food Sciences and Nutrition, Poznan University of Life Sciences, Wojska Polskiego 31, 60-624 Poznan, Poland; kprzybyl@up.poznan.pl; 2 Faculty of Food Sciences and Nutrition, Poznan University of Life Sciences, 60-624 Poznan, Poland; adaminaduda@wp.pl; 3 Institute of Biosystems Engineering, Poznan University of Life Sciences, Wojska Polskiego 50, 60-625 Poznan, Poland; 4 Department of Food Quality and Safety Management, Faculty of Food Science and Nutrition, Poznan University of Life Sciences, Wojska Polskiego 31/33, 60-624 Poznan, Poland; jerzy.stangierski@up.poznan.pl; 5 Main Library and Scientific Information Centre, Poznan University of Life Sciences, Witosa 45, 61-693 Poznan, Poland; mariusz.polarczyk@up.poznan.pl; 6 Faculty of Transport Engineering, Poznan University of Technology, Piotrowo 3, 60-965 Poznan, Poland; lukasz.gierz@put.poznan.pl

**Keywords:** Artificial Neural Networks (ANN), classification, strawberry, convection drying, acoustic signals, texture analysis

## Abstract

In this paper, the authors used an acoustic wave acting as a disturbance (acoustic vibration), which travelled in all directions on the whole surface of a dried strawberry fruit in its specified area. The area of space in which the acoustic wave occurs is defined as the acoustic field. When the vibrating surface—for example, the surface of the belt—becomes the source, then one can observe the travelling of surface waves. For any shape of the surface of the dried strawberry fruit, the signal of travelling waves takes the form that is imposed by this irregular surface. The aim of this work was to research the effectiveness of recognizing the two trials in the process of convection drying on the basis of the acoustic signal backed up by neural networks. The input variables determined descriptors such as frequency (Hz) and the level of luminosity (dB). During the research, the degree of crispiness relative to the degree of maturity was compared. The results showed that the optimal neural model in respect of the lowest value of the root mean square turned out to be the Multi-Layer Perceptron network with the technique of dropping single fruits into water (data included in the learning data set Z2). The results confirm that the choice of method can have an influence on the effectives of recognizing dried strawberry fruits, and also this can be a basis for creating an effective and fast analysis tool which is capable of analyzing the degree of ripeness of fruits including their crispness in the industrial process of drying fruits.

## 1. Introduction

The customer expectations and information included in the EU Commission Regulation changed the criteria of the quality evaluation of products. The approval of products has influence on, among other things, the looks [1], taste, aroma and texture [2], safety and nutritional values of products [3,4]. It is not allowed to accept an inadequate quality of fresh fruits, which can be, among other things, immature, overripe, moldy or too dirty. Consumers are becoming increasingly aware and are beginning to choose a balanced diet. They nourish themselves increasingly healthily, which leads to an increased demand for healthy snacks. An alternative to crisps, salty sticks or puff snacks can be dried fruits and vegetables. By selecting a proper method for the control of parameters during the drying process, products can keep their attractive sensory features and nutritional values. 

The fruit presented in the work is popular in Poland and in the world with regard to its sensory [5] and health [6,7] values. Strawberries are characterized by a pleasant sweet taste and firm, easy to consume pulp. They are consumed in the form of raw fruits. Strawberry preserves such as jams, mousses and sorbets are getting increasingly popular. It was also confirmed that strawberries have a positive influence on health, because they contain a complex of vitamin B, vitamin C and trace amounts of vitamin E and A, and micro-elements such as calcium and phosphorus [8,9]. They can be treated as a low-calorie snack, because 100 g of strawberries contain only 27 kcal. Strawberry contains anti-oxygen substances, which take part, among other things, in reducing the risk of cardiovascular diseases [9,10,11,12]. 

Dried fruits, which play a substantial role in the food industry, are becoming increasingly popular. Heat treatment processes, which are one of the most important methods of preserving the aforementioned food, ensure microbiological durability and reduce water activity by its elimination [13,14]. Water in food has a substantial influence on the safety, quality condition [15] and physical properties of food products [16], especially strawberry. Dried fruits must be free of contamination by, among other things, stones, insects and foreign bodies. Dried fruits can become only partially dried, burned or over-ripe as a result of chemical reactions [17,18]. 

In the age of technological development, techniques that support decision-making processes during production processes are being searched for. Acoustic signals have aroused interest [19], for example, in the analysis of acoustic wave shifts, which were used as a basic non-invasive method of evaluating the quality condition of watermelons. The effective analysis method and the low cost of the mentioned evaluation allowed the efficient determination of the quality class of watermelons with acoustic resonance. As a result of the conducted research, the effectiveness of identifying watermelon ripeness was determined at 95% [20]. Scientists noticed a substantial correlation between acoustic features and watermelon properties; among other things, the speed of acoustic wave dispersion seems to be lower as those fruits ripen [21]. Other experts proposed that the impulse of the acoustic spectrum reacted if there were any internal damage to the watermelons. It was determined that the adequate band of the amplitude spectrum frequency of the acoustic signal demonstrates the highest capability for detecting internal structure disorders [22]. 

During the research, the use of an acoustic signal to evaluate crispy or crunchy processed food, which includes dried fruits and vegetables, was taken into consideration. It has been found that food sound subject to external force has an influence on the material, causing deformation, damage or tearing. The release of the stored energy occurs when the research material cracks. Experts have evaluated the degree of carrot hardness on the basis of its firmness, and the results allowed carrot to be grouped according to the degree of hardness on account of positive and negative related acoustic waves [23].

The aim of this work was to draw up a classification method of dried strawberry fruits on the basis of parameters obtained from the acoustic spectrum, recognizing the degree of crispness and taking into consideration the degree of ripeness. In the research, two classes of dried strawberry fruits were used, derived from ripe and over-ripe fruits. The use of an acoustic signal was supported by machine learning in order to quickly evaluate the quality of dried strawberry juice in the production process, which is considered to be an innovation in the research.

## 2. Materials and Methods

### 2.1. Preparation of Samples

Fresh strawberries of the late Polish variety (Alfa Centauri) were obtained from a private company located in the Greater Poland region for the needs of the research. After the harvest, 2.5 kg of strawberries were selected to conduct the experiment. The research was conducted in the laboratory at the Institute of Food Technology of Plant Origin on the premises of Poznań University of Life Sciences. In the laboratory, experts evaluated the degree of strawberry ripeness (ripe, over-ripe) on the basis of their looks, color and taste (Table 1) [24]. Each of the distinguished features of quality under research (looks, color and taste) could reach 5 points; therefore, the combined value of evaluation, which allowed us to classify strawberry fruits, was up to 15 points. Strawberries which scored from 3 to 7 points were assigned to the over-ripe class, and those which scored from 8 to 15 points were classified as the ripe class. The indicated evaluation was determined on the basis of the norm that was strictly determined in the directive of the Commission of the European Communities on 21 May 2002.

### 2.2. Convection Drying

Another stage of the research required the determination of the weight and humidity of the research material. The mass of ripe and over-ripe strawberries was determined with laboratory scales with a precision of measurement of 0.001 g.

The humidity of strawberry fruits was determined with moisture analyzer type MA 30 (SARTORIUS). The precision of measurement of water content was 0.05%. Strawberry fruits were dried at 95 °C; i.e., the proper temperature for fruit products (PN-A-79011-3:1998). Three trials (repetitions) were conducted in the research.

**Figure 1:**
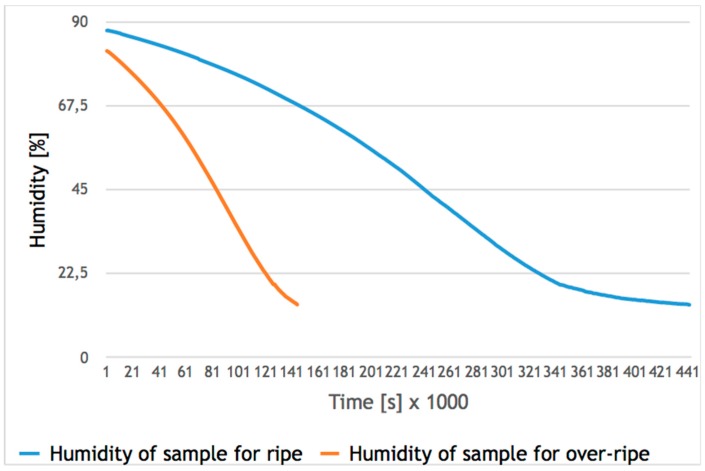
Convection drying curve for trials with ripe and over-ripe strawberry fruits in relation to the change in humidity over time.

The convection drying process of strawberry fruits (ripe and over-ripe) was carried out with a research stand [25,26]; the results of drying are presented in Figure 1. Fresh strawberry fruits were dried with convection drying at a temperature of drying factor of 60 °C, with a velocity of drying air flow of v = 1.0 m/s (controlled by means of an anemometer) and with parallel flow in a thin immobile layer.

The humidity of raw material was controlled during the whole process of drying with software that is used to back up the process of drying raw materials. After finishing the process, the final humidity of the raw material was verified with a moisture analyzer. The initial value of the humidity of the research material of the ripe class was 87.3%, and in the case of the over-ripe class, this equaled 82.7%. As a result of convection drying, the humidity of the research material was reduced to the level of 14% for both classes (Figure 1) [27].

### 2.3. Acoustic Signal Acquisition

**Figure 2:**
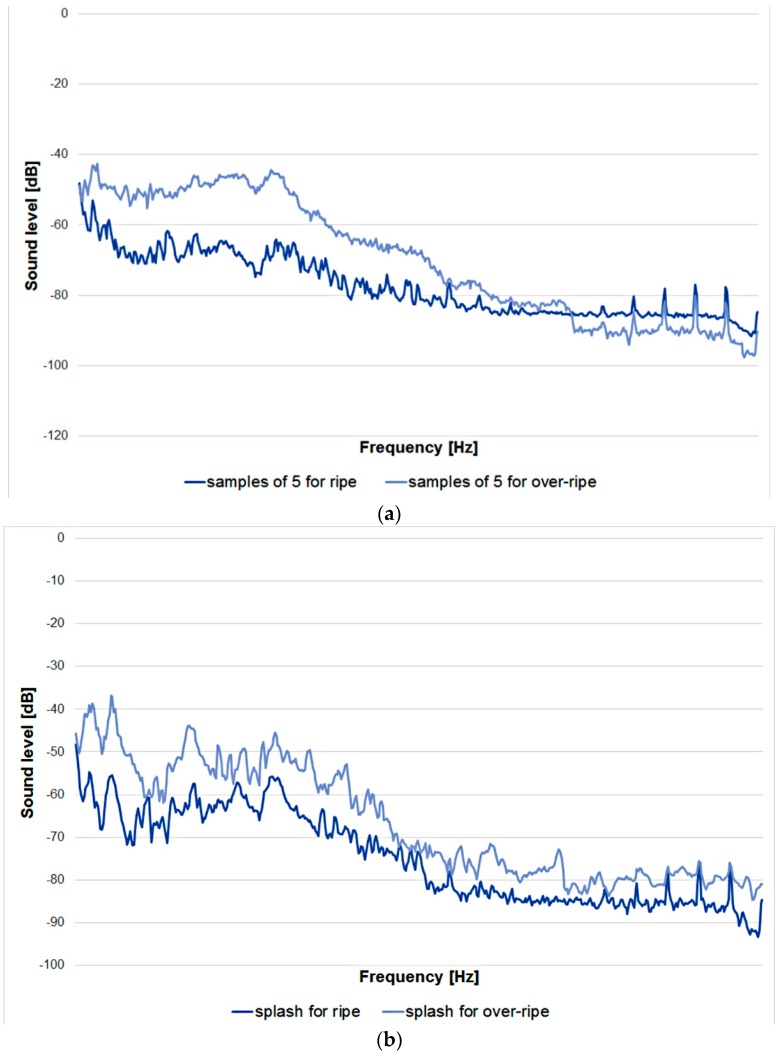

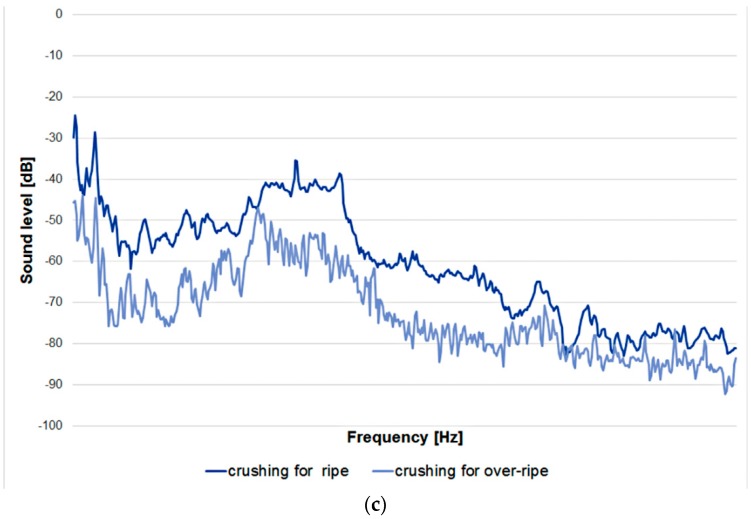
Samples of three acoustic spectra showing the level of sound intensity for dried strawberry fruits representing the ripe and over-ripe classes, with the following methods: (**a**) splash batch of five fruits, (**b**) splash batch of one fruit (the fruit lands in a glass filled with water), (**c**) squash batch with one fruit.

Within the research, a measurement and research stand were created in order to acquire the acoustic signal. The testing equipment of the stand consisted of, among other things, two pipes with a diameter of Ø 100 mm and lengths of 600 mm and 1000 mm, a test rack, and a trapdoor controlling the course of the speed of the drop of the research material. The selection of a proper microphone was an important source for the acoustic signal. The selected capacitor microphone was characterized by an acoustic impedance of 2.2 kW and frequency bandpass range of 50~16,000 Hz. On account of the characteristics of acoustic wave transfer, the research was done in a specially soundproofed studio designed for sound recording. Using the above type of room while recording the sound allowed us to avoid unnecessary outside noises. In the process of repeating the acquisition of the acoustic signal with the original testing stand, the calibration of parameters was conducted, among other things by adjusting the distance between the microphone and the pipe in order to avoid sound changes, up to the level of the so-called rumbling. The lower the difference of the acoustic wave interference, the less often the rumbling occurred. An essential parameter during the uninhibited drop of the research material was the determination of the proper inclination angle of the pipe (45°), with which it was possible to set the most effective speed and to avoid the phenomenon known as sliding. The last parameter—with the use of the trapdoor—allowed us to control the drop time of the research material. The process of the uninhibited drop of the dried strawberry fruit was conducted, among other things, in a batch of five fruits each. The characteristics of the acoustic wave are presented in Figure 2.

**Figure 3:**
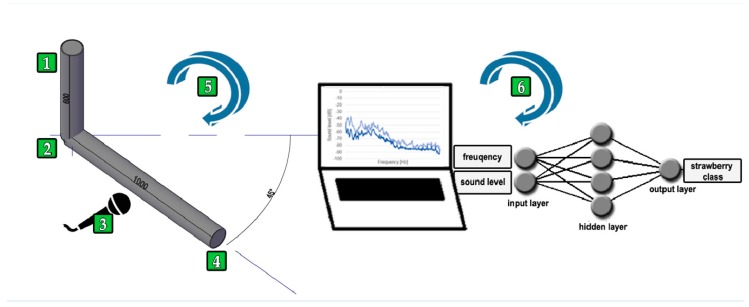
Scheme: 1–transport channel for dried strawberry fruit, 2–trapdoor controlling the speed of the uninhibited falling of fruits, 3–capacitor microphone, 4–dried fruit outlet dropping the dried fruit on the conveyor or into the glass filled with water, 5–import of the recorded sound to the PC and creation of the acoustic spectrum, 6–Artificial Neural Network (ANN) learning process with parameters determining the acoustic wave.

As part of the comparison of the way the longitudinal waves behaved, research was conducted with the original measurement and research stand (Figure 3) process of the uninhibited drop of dried fruits in a batch of one fruit each; this time, each fruit was dropped into a glass filled with water (Figure 2). In the process of repetition of the sound, the microphone changed location, and all the parameters were unchanged.

At the same time, one more investigation was conducted with the TA–XT2i texture analyzer, as a result of which basic parameters of the contact force were determined as well as the level of sound for the transverse wave. The acoustic spectrum is presented in Figure 2.

### 2.4. Texture Analysis of Dried Strawberry Fruit

**Figure 4:**
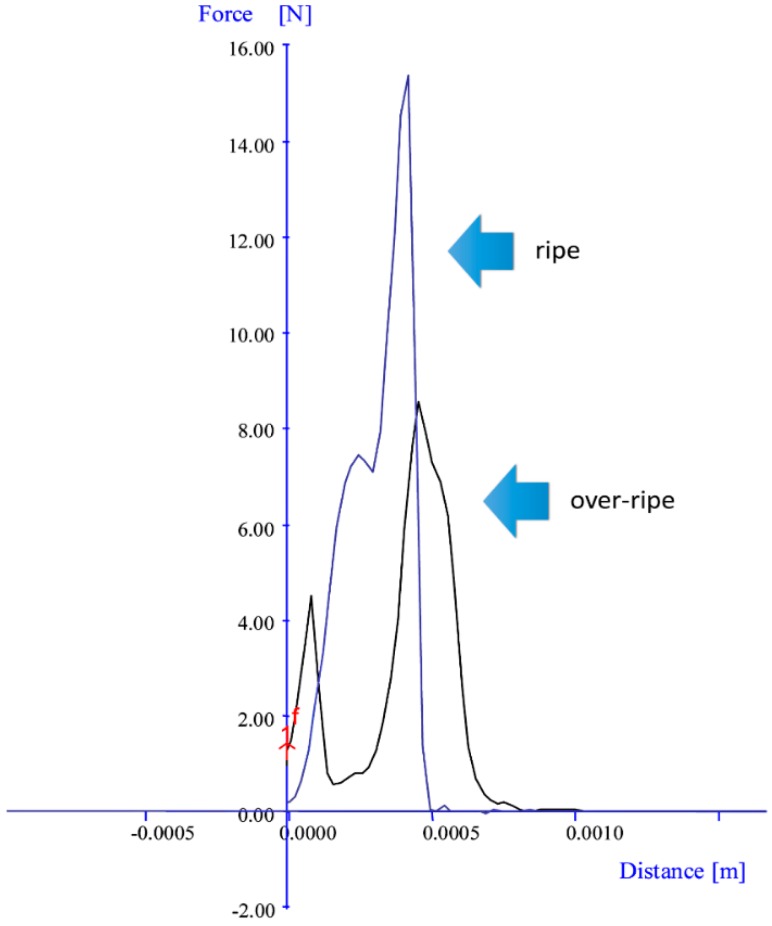
Graph of the relationship between strength and distance resulting from the texture of dried strawberry fruit and its degree of ripeness, namely ripe and over-ripe.

A test of the hardness of the dried strawberry fruits, taking into account their degree of ripeness with division into the ripe class and the over-ripe class, was also conducted (Figure 4). The research was conducted with the TA–XT2i Texture Analyzer (Stable Micro Systems, Godalming, Surrey, UK) [28]. One slice of the dried strawberry fruit with a total volume of mass of around 1 g was placed in the device. The microphone was put against the device in order to determine the frequency and the level of sound. The test of hardness was done in series of 20 repetitions for trials in the ripe and the over-ripe classes.

### 2.5. Structure of Training Sets

In the process of convection drying, 2.5 kg of strawberries were divided into three sets on account of the method of acoustic sound acquisition that was used. After processing the recordings, which were received with the free software Audacity (https://www.audacityteam.org/), numerical data of the acoustic spectrum were extracted, namely the frequency (Hz), and the level of sound intensity (dB). The structure of the training sets consisted of series of learning cases representing acoustic spectrums (Figure 2) in the form of two input variables and of expert opinion determining trials of the dried strawberry fruits for the ripe and the over-ripe class in the form of one nominal (two-state) output variable. The first learning set (Z1) regarded the splash series of five fruits, and 120 cases were obtained (60 in the ripe class and 60 in the over-ripe class). The second set (Z2) was created for the batch of strawberries that were dropped one by one and landed in water, which allowed us to obtain 40 cases (20 cases in the ripe class and 20 cases in the over-ripe class). The last set (Z3) was created for the fruit that was crushed individually with the TA–XT2i texture analyzer, as a result of which 40 cases were obtained with the division of the dried strawberry fruit as in the second set. The learning sets were created in a spreadsheet (MS Excel) and then were imported to the software Statistica. Each set represented measurement representative characteristics divided into a 2:1:1 ratio and then into subsets: training, validating and testing (which were not used in the process of generating classificatory neural models).

### 2.6. Preparation of Artificial Neural Networks

Artificial Neural Networks (ANN) are an artificial tool which allows us to reproduce complicated dependencies between neatly selected input variables and well-defined output variables [29,30]. As one of the methods of artificial intelligence, they arouse interest in an ever-growing number of fields dealing with problems and issues; among other things, ANN can be used in automating current manual processes including in agricultural and food sectors [31,32,33]. Using them in intelligent information systems to perform analysis and recognition with sound or images simplifies production processes, the systematic monitoring of processes and the control of production process as well as the quality evaluation of finished goods. The research work that was done is one the examples of using ANN [2,34,35] which aims, among other things, at automating the process of controlling the quality condition of dried fruits with acoustic signals.

In the research paper, the artificial neural network Multi-Layer Perceptron (MLP) 2: 2-6-1:1, was created on the basis of two neurons in the input layer, six neurons in the hidden layer and one neuron in the output layers. The MLP 2:2-14-1:1 network consisted of two neurons in the input layer, 14 neurons in the hidden layer and one neuron in the output layer. The MLP 2:2-4-1:1 network was created with two input variables, four neurons in the hidden layer and one output variable.

The MLP 2:2-6-1:1 network was characterized by the quality ratio for learning [36] at a level of 0.96 for the training set, the testing set and the validation set (Table 2), as well as a mean square error (MSE) [37] at a level of 0.03 (Table 3). The MLP 2:2-14-1:1 network reached the highest classification ratio at a level of 0.98 (Table 2) and MSE at a level of 0.09 (Table 3). The last MLP 2:2-4-1:1 network reached the lowest quality of training in relation to the two remaining models, and its classification ratio was 0.82 (Table 2) and its MSE was 0.12 (Table 3).

The Back-Propagation (BP) algorithm was presented in the process of learning, namely the learning function using a reverse propagation of error [38]. MLP 2:2-4-1:1, for the first stage of learning with BP, reached the lowest level of error in the sixth learning set, whereas the MLP 2:2-6-1:1 network and the MLP 2:2-14-1:1 network, in the first stage of learning with BP, reached the lowest level of error in the 50th learning set. Another stage of learning for the MLP 2:2-14-1:1 network and the MLP 2:2-6-1:1 network was guaranteed by the conjugate gradient algorithm (CG) [39,40], for which the lowest level of error was reached in the 19th and the 134th iteration, respectively.

## 3. Results and Discussion

### 3.1. Classification

Hundreds of simulations were made with the ANN simulator in Statistica, as a result of which three adequate neural networks were chosen (Table 2). Networks characterized by the greatest ability for classification were defined by the Multi-Layer Perceptron (MLP) model [41,42,43]. This type of network turned out to be appropriate for the batch of five fruits, with the sound of fruit falling into the water and the sound of the crushed strawberry. Input data, which differ in terms of the quantity in each batch (“five fruits”, “splash”, “crushing”), inform us about the representative characteristics of models encoded in sound. Individual structures also differ in terms of the number of neurons in the hidden layer (in the batch with five fruits, in the batch of the fruit falling into the water and in the batch characterized by the sound of the crushed strawberry). Input variables of all batches are recognized on the basis of numeral data obtained from the acoustic wave and after assigning them to the proper output variable: either the ripe or the over-ripe class. The Artificial Neural Networks that were created met the classification requirements. The best classification results within the range of networks using the sound of the strawberry falling in an uninhibited manner were obtained in the “splash” batch, which was characterized by the highest quality of learning and testing.

Equally good results were achieved during the simulations of the batch with the uninhibited falling “of five fruits”. Research into the uninhibited falling of the batch “of one fruit” and in the batch “of 10 fruits” was done as part of the comparison. The results of the neural model training in relation to the aforementioned batches were not satisfying. In the batch “of one fruit”, there was a high training error, and in the batch “of 10 fruits”, the phenomenon of network overtraining occurred.

A sequence of simulations was conducted in the “crushed” batch. It fulfilled its goal with satisfying results, with a slightly worse result than the best network created for the batch using the sound of the uninhibited falling of the strawberry. The quality of learning in all the created networks was higher than quality of testing, which means that the network is characterized by good quality. A higher value of the quality of network learning means that the network is capable of generalizing and is not over trained.

Using methods with the acoustic signal as a result influenced the selection of the neural network for the evaluation of dried strawberry fruit for one of the classes: either the ripe class or the over-ripe class. The created networks fulfilled their task, namely the classification of the quality of dried strawberry fruit on the basis of the sound of the strawberry falling in an uninhabited manner or on the basis of the sound of a strawberry that was crushed. MLP 2:14:1 had the highest classification ability without making mistakes, which was created for the learning set using the sound of the strawberry that fell into a little tank with water in an uninhabited manner; the quality of learning was close to 1, which means that this network was of very high quality. Network MLP 2:6:1 had satisfying results; this network was created on the basis of the learning set using the sound of the strawberry falling into the water in the batch with five fruits in each batch. Network MLP 2:4:1 had the lowest results and was created on the basis of the set based on the sound of the crushed strawberry with a texture analyzer; despite achieving lower results for the parameters of quality of learning and quality of testing, this network was cable of classifying the strawberry class almost infallibly.

### 3.2. Validating the Artificial Neural Networks

After completing the process of learning, the validation of the selected neural models was conducted (Table 2). Each model was evaluated with the mean absolute deviation (MAD) by using Equation (1), the mean absolute percentage error (MAPE) by using Equation (2), the mean square error (MSE) by using Equation (3) and the root mean square error (RMSE) by using Equation (4) [43]. MAPE is the average of absolute errors divided by actual observation values. MSE is probably the most commonly used error metric [44,45]; it penalizes larger errors, because squaring larger numbers has a greater impact than squaring smaller numbers. MAD is the sum of absolute differences between the actual value and the forecast divided by the number of observations [46,47,48].

As a result of the learning process, and after conducting the validation, it was seen that the selected models were characterized by the lowest error value of RMS as well as a low value of MAPE, oscillating in the region of about 20%. The results (Table 3), in which RMSE and MAPE reached a value of about 10%, mean that the model performs well in the process of dried strawberry fruit identification [45,48].

(1) $$\mathrm{MAD}=\frac{\sum_{i=1}^{n}\left|y_{i}-z_{i}\right|}{n},$$

(2) $$\mathrm{MAPE}=\frac{\sum_{i=1}^{n}\left|\frac{y_{i}-z_{i}}{y_{i}}\right|}{n}\times 100,$$

(3) $$\mathrm{MSE}=\frac{\sum_{i=1}^{n}{\left(y_{i}-z_{i}\right)}^{2}}{n},$$

(4) $$\mathrm{RMSE}=\sqrt{\frac{\sum_{i=1}^{n}{\left(y_{i}-z_{i}\right)}^{2}}{n}},$$

where n is the number of cases; y_i_ is the real value; and z_i_ is the value determined with ANN.

### 3.3. Texture Analysis

Research results for product hardness for the individual classes are presented in Table 4. The level of crispness for food products—i.e., snacks—has a substantial meaning for consumers. It turned out that, after completing the process of convection drying and while keeping the contact humidity value at the level of 14% for both the classes, the ripe fruits reached a level of hardness of 15 N on average. This is twice as large as the strength in the case of the over-ripe fruits, which could mean that there was higher humidity in the dried strawberry fruit in trials of the ripe class than in the dried strawberry fruit in trials of the over-ripe class. The differences in the values of strength in trials of the ripe fruits in relation to trials of the over-ripe fruit—which were obtained with a texture analyzer—show the classified trials of the ripe batch are characterized by a higher degree of crispness than the classified trials of the over-ripe batch of dried strawberry fruit (Figure 4).

## 4. Conclusions

Neural networks, which are part of artificial intelligence, are increasingly being used to support the optimization, control or implementation of different production processes. They allow the complete automatization and control of decision-making processes in food production. The research demonstrated that the MLP 2:14:1 for the Z2 type, which obtained a RMS error at the level of 0.09 and MAPE at the level of about 10%, is characterized by the highest classification ability. The printed neural model determined that the second method allows a better recognition of quality condition in dried strawberry fruit as opposed to the other methods.

The use of acoustic sound backed up by the method of machine learning allowed the fast and effective recognition of the class of research material, also with respect to its level of crispness with different quality conditions. The acoustic signals can support the evaluation of the quality condition of dried strawberry fruit, as conducted in the case of other products; among other things, watermelons or carrots. They can also serve as an objective evaluation of crispness. Using acoustic signals can also eliminate human error, which is usually due to individual preferences or tastes disturbing the objective values of the crispness of products.

In the described research on drying strawberry fruits, it was also noticed that, on account of the difference of the initial humidity value in the two trials of the material, an unfavorable change in color, texture and shape in the trials of the over-ripe class was observed. Changes observed in the ripe strawberries were much less distinct. Drying is one of the safest methods of preserving food. This method is very popular because of the simplicity of building dryers. Thanks to a wide variety of convection dryers, one can dry almost any product with this device, irrespective of the shape or state of matter (e.g., whole fruits, juice, granules, or paste).

## Figures and Tables

**Table 1:** Criterion for strawberry evaluation conducted by experts.

Quality Ratio	1 Point	2 Points	3 Points	4 Points	5 Points
Looks	Very soft fruit with numerous stewed areas on the surface	Soft fruit with visible stewed areas on the surface	Firm fruit without deformations with light stewed areas on the surface	Firm fruit without deformations, lack of visible stewed areas on the surface	Hard fruit
Color	Red, numerous dark red marks	Red with dark red marks	Dark red	Red	Light red
Taste	Strongly perceptible alcohol aftertaste	Sweet with detectable alcohol aftertaste	Characteristic of strawberry, intense sweet	Characteristic of strawberry, sweet	Characteristic of strawberry, sweet and sour

**Table 2:** Network structure created on the basis of the learning sets prepared for dried strawberry fruit. BP: Back-Propagation; CG: conjugate gradient algorithm.

Name	Z1	Z2	Z3
Model ANN	MLP 2:2-6-1:1	MLP 2:2-14-1:1	MLP 2:2-4-1:1
Training error	0.14	0.01	0.32
Validation error	0.04	0.09	0.29
Testing error	0.32	0.18	0.45
Quality of learning	0.98	0.98	0.85
Quality of validation	0.99	0.99	0.86
Quality of testing	0.90	0.96	0.76
Learning cases	120	40	40
Training algorithm	BP50, CG134b	BP50, CG19b	BP06

**Table 3:** The results of validation for the strawberry neural models. MSE: mean square error; RMSE: root mean square error; MAD: mean absolute deviation; MAPE: mean absolute percentage error.

Name	Model ANN	MSE	RMSE	MAD	MAPE
Z1	MLP 2:2-6-1:1	0.03	0.16	0.36	20.55
Z2	MLP 2:2-14-1:1	0.01	0.09	0.19	10.39
Z3	MLP 2:2-4-1:1	0.12	0.35	0.71	5.57

**Table 4:** Hardness (N) of the selected strawberry classes obtained with TA–XT2i texture analyzer.

No.	Ripe Class (N)	Over-Ripe Class (N)
1	13.1	10.1
2	15.6	12.4
3	16	9.9
4	13.6	8.8
5	18.2	7.3
6	15.6	7.6
7	12.3	9.1
8	15.4	8.7
9	12.3	8.3
10	15.4	6.9
11	17.8	7.1
12	12.9	7.1
13	14.4	10.1
14	14.6	7.3
15	12.5	8.6
16	14.6	6.8
17	18.3	6.9
18	14.8	10.1
19	12.9	10.9
20	14.6	9.3
**Mean**	**15**	**8.7**
